# An Open-Source Algorithm for Correcting Stress Wave Dispersion in Split-Hopkinson Pressure Bar Experiments

**DOI:** 10.3390/s25010281

**Published:** 2025-01-06

**Authors:** Arthur Van Lerberghe, Kin Shing O. Li, Andrew D. Barr, Sam D. Clarke

**Affiliations:** School of Mechanical, Aerospace & Civil Engineering, University of Sheffield, Sheffield S1 3JD, UK

**Keywords:** signal processing, dispersion correction, high-strain-rate testing, stress waves, split-Hopkinson pressure bar, material applications, open-source algorithm

## Abstract

Stress wave dispersion can result in the loss or distortion of critical high-frequency data during high-strain-rate material tests or blast loading experiments. The purpose of this work is to demonstrate the benefits of correcting stress wave dispersion in split-Hopkinson pressure bar experiments under various testing situations. To do this, an innovative computational algorithm, SHPB_Processing.py, is created. Following the operational run through of SHPB_Processing.py’s capabilities, it is used to process test data acquired from split-Hopkinson pressure bar tests on aluminium, sand and kaolin clay samples, under various testing conditions. When comparing dispersion corrected and simple time shifting data obtained from SHPB experiments, accounting for dispersion removes spurious oscillations and improves the inferred measurement at the front of the specimen. The precision of the stress and strain results gathered from its application emphasises its importance through the striking contrast between its application and omission. This has a significant impact on the validity, accuracy and quality of the results. As a result, in the future, this tool can be utilised for any strain rate testing situation with cylindrical bars that necessitates dispersion correction, confinement, or stress equilibrium analysis.

## 1. Introduction

Traditionally, a Hopkinson pressure bar (HPB) is used to quantify a transitory pulse generated by the impact of near-field blast events or bullets. The split-Hopkinson pressure bar (SHPB), also known as the Kolsky bar, has been widely utilised to measure dynamic material properties such as stress–strain and strain rate–strain curves of versatile materials at a strain rate ranging from 10^2^ to 10^4^ s^−1^. The shape of the elastic wave in SHPB and HPB distorts as it travels; this phenomenon is referred to as dispersion [1].

From the standpoint of medium particle motion, the physical origin of dispersion is inertia in the lateral motion associated with the axial disturbance. From the standpoint of wave propagation, a high-frequency wave component that constitutes the total elastic wave is slower than a lower-frequency wave component [2].

The wave profile is typically assessed at the interim axial position of the bar, using strain gauges. In the case of the HPB, the front surface of the bar is the location of interest where an impact pulse enters the bar, whereas in the case of the SHPB, the specimen location is of interest. Consequently, the measured wave profiles in HPB and SHPB must be corrected to obtain the wave profiles at the locations of interest, a procedure known as dispersion correction [1,3].

The one-dimensional wave theory assumes that all longitudinal waves in the bar travel at a constant velocity, $c_{0}$. It also assumes that transverse cross sections of the bar remain flat and that stresses are uniformly distributed across these sections. However, as the wave moves along the bar, it causes radial expansion and contraction due to axial strains, influenced by the bar’s Poisson’s ratio. This radial motion disturbs the stress distribution across the bar’s cross section, resulting in the distortion of plane sections [2].

The effect of this deviation from the idealised conditions is evident in the three-dimensional wave equations developed by Pochhammer [4] and Chree [5], which were later applied by Bancroft [6] to longitudinal waves in a cylindrical bar. Instead of propagating uniformly at a velocity $c_{0}$, longitudinal waves were shown to propagate at a specific velocity $c_{\omega }$, which depends on the wavelength, the bar’s diameter, the one-dimensional wave speed, and Poisson’s ratio as described in Equation (1):

(1) $${\left(x-1\right)}^{2}\varphi \left(ha\right)-\left(\beta x-1\right)\left[x-\varphi \left(\kappa a\right)\right]=0$$

where

$$\begin{array}{cc} \beta & =(1-2\nu )/(1-\nu )\\ x & ={\left(c_{\omega }/c_{0}\right)}^{2}\left(1+\nu \right)\\ h & =\gamma {\left(\beta x-1\right)}^{\frac{1}{2}}\\ \kappa & =\gamma {\left(2x-1\right)}^{\frac{1}{2}}\\ \varphi (y) & =yJ_{0}\left(y\right)/J_{1}\left(y\right) \end{array}$$

and where $c_{\omega }$ is the phase velocity, $c_{0}$ is the one-dimensional elastic wave velocity, *a* is the bar radius, ν is the Poisson’s ratio, γ is the wave number, 2π/λ, λ is the wave length, and $J_{n}$( ) is the Bessel function of the first kind, of the order *n*.

**Figure 1:**
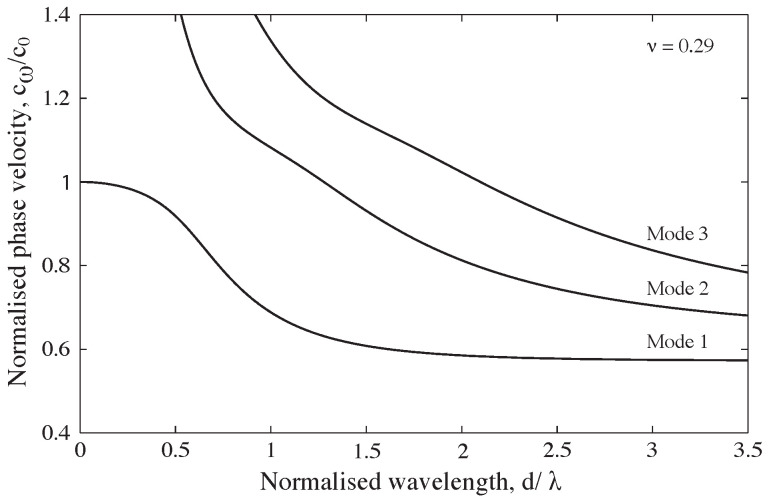
Relationship of phase velocity to wavelength for the first 3 modes of propagation of a longitudinal wave in a stainless-steel cylindrical bar for ν = 0.29 [7].

This equation has an infinite number of solutions, each corresponding to a specific propagation mode in the bar, with the first modes illustrated in Figure 1. This implies that low-frequency waves propagate at a velocity approximately equal to $c_{0}$, but the phase velocity decreases as the frequency increases, particularly when the wavelength approaches the bar’s diameter.

**Figure 2:**
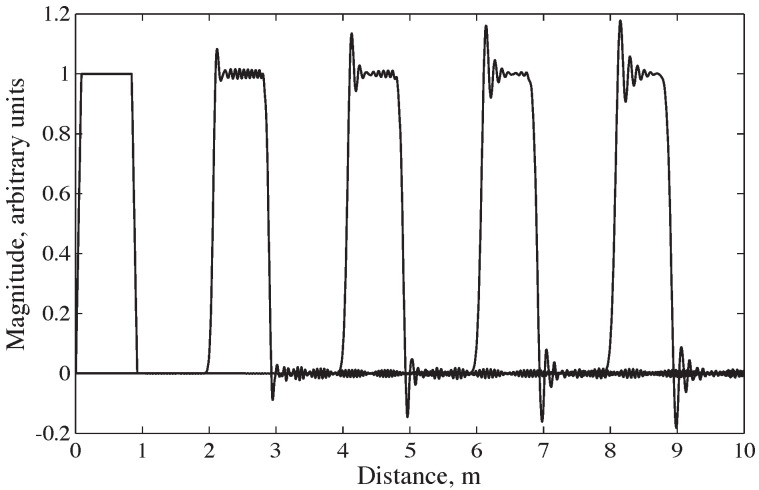
Dispersion of a trapezoidal wave in a cylindrical stainless-steel pressure bar, with recordings at 2 m increments [7].

**Figure 3:**
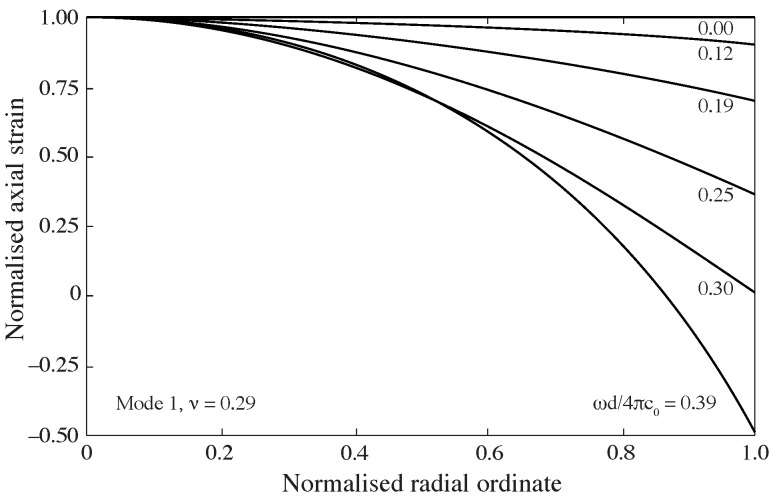
Distribution of axial strain over a stainless-steel (ν = 0.29) bar cross section for an infinite duration single frequency forcing function [7], after [11].

The complex waveforms generated during an SHPB experiment encompass a broad range of frequency components. Due to this frequency dependence, stress disperses as it travels along the bar. This phenomenon is illustrated in Figure 2, which shows the dispersion of a trapezoidal wave in a stainless-steel pressure bar. The dispersion of the stress pulse is accompanied by frequency-dependent variations in stress and strain across the bar’s cross section [7]. As shown in Figure 3, when the frequency of the forcing function increases, the strains recorded on the bar surface become smaller compared to those measured at the bar’s axis. These effects on phase velocity and amplitude mean that a strain signal recorded at the surface of the bar may not accurately represent the mean strain and stress at the bar face in contact with the specimen, some distance away.

Standard practice, as discussed by Gray III [8] in the ASM handbook, assumes that simply time shifting all the signals collected from SHPB testing is a suitable strategy; however, this method can result in severe errors and inaccuracies.

Previous work by Shin [1,3,9] developed dispersion-related MATLAB and Excel scripts to process SHPB test data. These algorithms focused on phase velocity corrections but not amplitude correction. While useful for many applications, experiments with high-frequency components, or a large diameter, will experience significant stress and strain variation over the bar cross section, making amplitude correction desirable for accurately evaluating specimen behaviour [10].

The current work seeks to develop an algorithm capable of solving the issues associated with dispersion in SHPB experiments. To accomplish this, the key theory of dispersion correction, stress wave equilibrium and confinement analysis in SHPB experiments is addressed first. Then, the aforementioned tool, SHPB_Processing.py, is presented with all its functionalities and subroutines. Finally, it is applied to SHPB test data collected, demonstrating its practical importance.

## 2. Dispersion Correction in SHPB Experiments

At higher frequencies (a/λ> 0.05), the errors mentioned above become considerable, but they can be addressed using the method outlined by Tyas and Pope [10], where corrections are applied to the amplitudes and phase angles of each frequency component of the signal.

### 2.1. Phase Angle Correction

The first correction made to the SHPB signals is the adjustment of the phase angle to account for the dispersion of each frequency component over the distance between the strain gauge and the bar end. This is accomplished, according to Gorham [12] and Follansbee and Frantz [13], by computing the phase velocity $c_{\omega }$, of each component using Bancroft’s [6] equation (Equation (1)) and then applying a phase shift, $\theta_{\omega }^{\prime }$ as portrayed in Equation (2):

(2) $$\theta_{\omega }^{\prime }=\left(\frac{c_{0}}{c_{\omega }}-1\right)\frac{\omega z}{c_{0}}$$

where ω is the component’s angular frequency, and *z* is the distance over which the correction is performed, positive in the direction of wave propagation.

Barr et al. [14] conducted tests to understand how energy is distributed between higher modes of propagation, concluding that the frequency content in common SHPB experiments only requires consideration of the first mode of propagation.

### 2.2. Amplitude Correction

The second correction to the SHPB signals involves applying factors to the amplitude of the frequency components. Tyas and Watson [11] established the factors $M_{1}$ and $M_{2}$ to account for the radial variation of strain and Young’s modulus, respectively, derived from Davies’ [15] investigation of these radial effects. Using these factors, the strain measurement obtained on the bar’s surface can be utilised to calculate the mean axial stress and strain acting over the entire cross section. In a SHPB experiment, phase angle (dispersion) correction transforms a bar surface measurement at the strain gauge to a bar surface measurement at the specimen interface; the amplitude correction transforms this bar surface measurement into the mean strain and stress experienced across the face of the specimen.

The factors are defined in Equations (3) and (4) as follows:

(3) $$M_{1}=\frac{2\left(1+\frac{1-\beta x}{x-1}\right)}{\varphi \left(ha\right)+\frac{1-\beta x}{x-1}\varphi \left(\kappa a\right)}$$

(4) $$M_{2}=E{\left(\frac{c_{\omega }}{c_{0}}\right)}^{2}$$

where details of the variables in Equations (3) and (4) are the same as in Equation (1), with *E* being the Young’s modulus.

**Figure 4:**
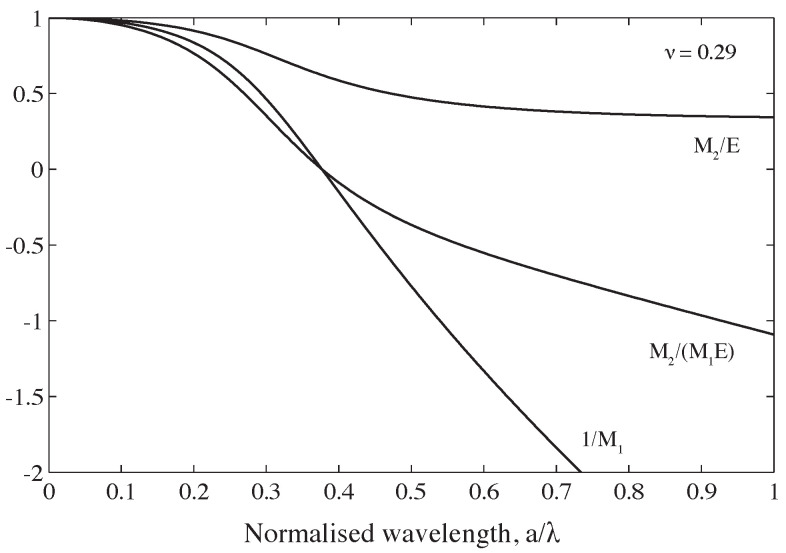
Variation in factors $M_{1}$ and $M_{2}$ in a cylindrical stainless-steel bar for ν = 0.29 [7].

Figure 4 shows the fluctuation in $M_{1}$ and $M_{2}$ with normalised wavelength for a stainless-steel bar with a Poisson’s ratio of 0.29. Due to the discontinuity in $M_{1}$ at a/λ = 0.375, which corresponds to the point where the strain recorded on the surface of the bar falls to zero, the reciprocal of $M_{1}$ is displayed; at even higher frequencies, the recorded strain has the opposite sign to the mean cross-sectional response. As the adjustments applied at a/λ = 0.375 require multiplying a low-magnitude signal by a very large correction factor, noise in the signal is likely to affect the accuracy of the result significantly. This effectively establishes an upper limit on the frequency range that can be corrected: according to Tyas and Watson [11], the approach can be used at normalised wavelengths below a/λ≈ 0.3.

## 3. SHPB_Processing.py

SHPB_Processing.py is an open-source Python algorithm for high-strain-rate SHPB signal processing. This function includes a subroutine titled dispersion.py that is optimised to process raw signal strain data using dispersion correction (Section 4).

This function, SHPB_Processing.py, is designed to take strain gauge input data from high-strain-rate SHPB tests and, by specifying the additional input variables defined in Table 1, determine the axial and radial (if confinement specified) stress developments of the sample, its strain and strain rate history variations through impact, and other related parameters derived from these output variables.

The following command line is necessary to run this algorithm:

SHPB_Processing(csv_path,sample_data,confinement,signal_channels,

      signal_amp,disp_correction,alignment,speedtrap)

**Figure 5:**
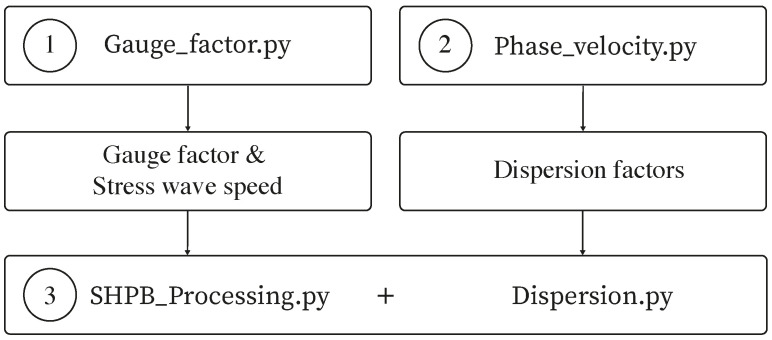
Flowchart illustrating the steps to run SHPB_Processing.py efficiently.

The optimal approach to running this function is detailed below, with Figure 5 depicting this as a concise flowchart:

1. Calculate stress wave speed and gauge factors of the cylindrical bars used for SHPB testing using the gauge_factor.py script, available on GitHub and ORDA [16].
2. Use phase_velocity.py to calculate the dispersion factors required to perform the dispersion correction of the collected SHPB signals using dispersion.py based on the material properties of the cylindrical bar used for SHPB testing. The algorithm phase_velocity.py is available on GitHub and ORDA.
3. The algorithm SHPB_Processing.py is ready to be run, with dispersion.py, to effectively process the SHPB test data with dispersion correction, based on the input parameters chosen. The results are returned in a designated processed data folder. Dispersion.py is available on GitHub and ORDA.

The full source code for SHPB_Processing.py is available on GitHub and ORDA [17].

The function’s operation can be summarised as follows:

1. The oscilloscope data from SHPB strain gauges are read.
2. The striker bar velocity is determined based on the raw speed trap data.
3. The raw data file is prepared for correction and confinement analysis via pulse detection and signal reformatting.
4. The correction (‘True’ for dispersion correction, or ‘False’ for simple time shift) and confinement (‘None’, ‘Ring’ or ‘Reservoir’) requirements are applied on the strain data collected based on the input specifications.
5. The incident, reflected, and transmitted pulses are detected using the trigger and wave speed propagation in the bars used during SHPB testing.
6. The pulse end is marked when the sample strain reaches its maximum.
7. The dispersion-corrected stresses and strains for each wave are calculated using dispersion.py, the details of which are present below. For simple time shifting, simple signal restructuring is conducted.
8. The axial stresses and strains in the specimen are calculated using the incident, reflected, and transmitted wave signals.
9. Based on the strain gauge strain, the sample strain is determined from the displacement of the pressure bars.
10. Based on the confinement type selected, ‘None’, ‘Ring’, or ‘Reservoir’, the following will happen:
  (a) For a SHPB test with ‘None’ as the confinement type, no radial stresses or strains are calculated for the specimen.
  (b) For a SHPB test with ‘Ring’ as the confinement type, using thick-walled pipe theory, the radial stress and strain in the specimen are calculated from the circumferential strain in the ring.
  (c) For a SHPB test with ‘Reservoir’ as the confinement type, pressure data collected from the gauge in the reservoir are used to calculate the specimen’s radial stress and strain.
11. The specimen density and dry density are calculated for the ‘Ring’ and ‘Reservoir’ confinement types.
12. All results are saved as csv files into the Processed data folder, along with the test log.

## 4. Dispersion.py

### 4.1. A Python Function for Dispersion Correction

Dispersion.py is an open-source Python algorithm that has been developed to automate the application of phase-angle and amplitude corrections to SHPB signals as part of the main processing of SHPB_Processing.py. This substitutes basic time shifting of signals with manipulation of individual frequency components. The capabilities of this function are described in this section, with the complete source code for dispersion.py and its accompanying subroutine available on GitHub and ORDA [18].

### 4.2. Frequency Domain in Python

The fast Fourier transform (FFT) is an algorithm used to convert a signal into the frequency domain. This technique portrays a signal as the sum of a sequence of sinusoidal waves of varying frequencies and amplitudes. FFT is implemented in Python using the numpy library and fft function, which takes any regularly sampled signal and returns amplitude and phase information with frequency as a matrix of complex vectors of the form *z* = *zr* + *iz_i_ *. At a given frequency, amplitude *A* (Equation (5)) and phase angle θ (Equation (6)) of the Fourier component are calculated as

(5) $$A=\sqrt{z_{r}^{2}+z_{i}^{2}}$$

(6) $$\theta =\tan^{-1}\left(\frac{z_{i}}{z_{r}}\right)$$

**Figure 6:**
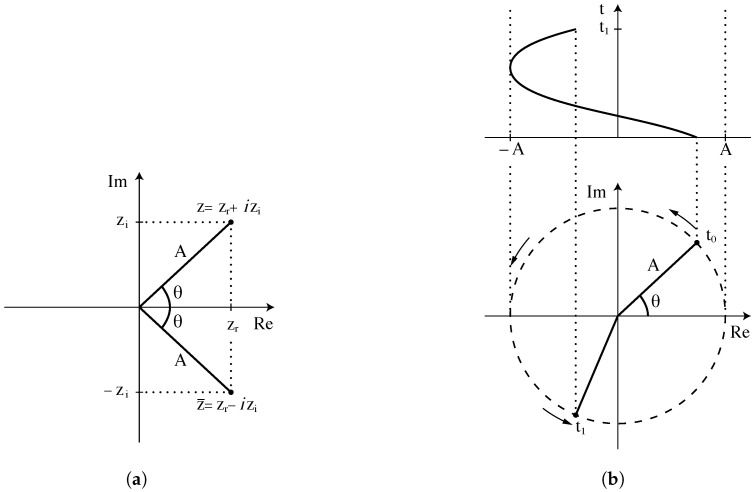
A Fourier component *z* in the complex plane with (**a**) relationship to amplitude and phase angle and (**b**) description of a sinusoid [7].

These relationships are illustrated in Figure 6a, where *z* and its complex conjugate $\bar{z}$ are represented in the complex plane, and in Figure 6b, where these values are utilised to represent the amplitude and phase angle of a specific sinusoid.

The Fourier component can be reconstituted using the relationship in Equation (7) once suitable corrections have been applied to the amplitude and phase angle as seen below:

(7) $$z=A\cos\left(\theta \right)+iA\sin\left(\theta \right)=Ae^{i\theta }$$

### 4.3. Correction Bandwidth

**Figure 7:**
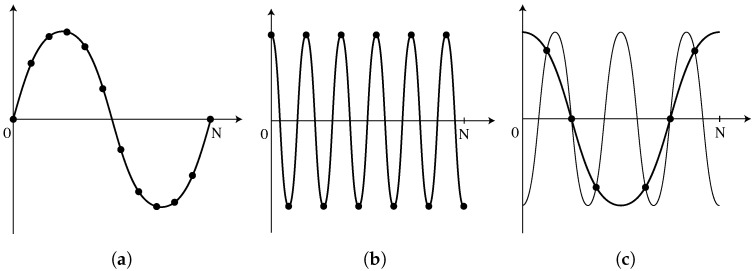
Frequency limitations in the FFT: (**a**) minimum readable frequency, where a single wavelength occupies the full sampling window, (**b**) maximum readable frequency, with only two samples per period, and (**c**) aliasing at higher frequencies, where multiple sinusoids can fit the same data [7].

The FFT is an algorithm to efficiently compute the discrete Fourier transform (DFT) of a signal. The DFT calculates frequency components at a finite number of values, which depend on the original signal’s sampling rate and length. If a signal is sampled *N* times at a frequency *f*, the lowest readable frequency is equal to *f/N*, describing a single wave occupying the sampling window (Figure 7a). Higher frequencies are multiples of the fundamental frequency, all the way up to the highest readable frequency, or Nyquist frequency, which equates to *f*/2 (Figure 7b). This limit is set because at least two samples are necessary for each period to prevent aliasing as shown in Figure 7c. Due to undersampling, two different sinusoids can be fitted to the sample data. The oscilloscope’s sample rate (*f*/2 = 500 kHz in the current tests) limits the highest readable frequency, although the frequency resolution can be improved by raising *N*, either by increasing the recording duration, or by zero-padding the input signal.

**Figure 8:**
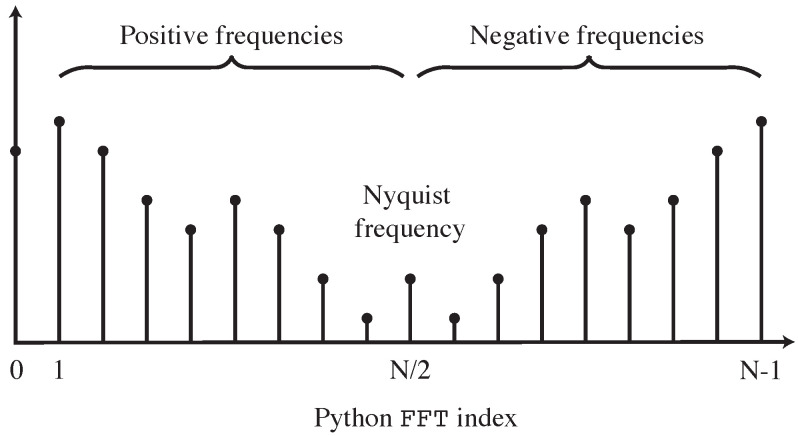
Composition of the frequency domain vector produced by fft in Python. The highest readable frequency is the Nyquist frequency, N/2. The second half of the vector represents the complex conjugate of the values in the first half [7].

The fft function will generate an *N*-length frequency domain vector *X*(ω), given an *N*-length time-domain vector *x(t)*. As a result of the aliasing explained above, the second half of *X*(ω) is the complex conjugate of the first half, reflected about the Nyquist frequency as seen in Figure 8. This means that modifications only need to be individually applied to the first *N*/2 + 1 bins in *X*(ω), which may then be reflected to complete the vector.

**Figure 9:**
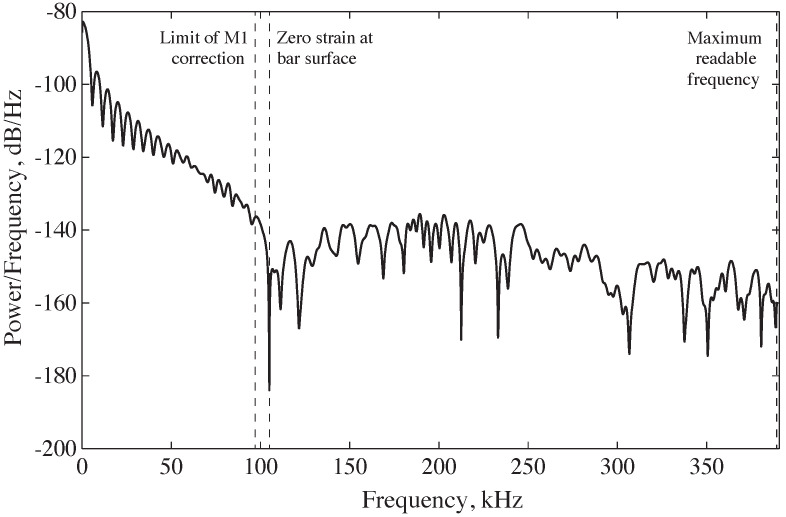
Power spectral density for experimental incident wave, from a 25 mm stainless-steel bar with a Poisson’s ratio of 0.29, and maximum frequency limits imposed by the strain gauge data and FFT [7].

As stated in Section 2.2, the very low strain signals measured on the surface of the bar at wavelengths below a/λ≈ 0.3 impose an additional frequency limit. For example, for a 25 mm diameter stainless-steel bar, adjustments can only be successfully made between 39 µHz and 94 kHz in the current SHPB setup. Figure 9 depicts a frequency domain portrayal of a typical experimental incident pulse in the form of a modified periodogram. Power is measured in logarithmic units, with a charge of 10 dB denoting an order of magnitude shift in the power of the signal. The periodogram, as explained above, indicates that the power of the signal recorded on the surface of the bar rapidly decreases to zero between 94 kHz and 110 kHz. Since dispersion correction can only be implemented at frequencies below 94 kHz, for this setup, the signal is sent through a low-pass filter to remove the higher frequencies.

**Figure 10:**
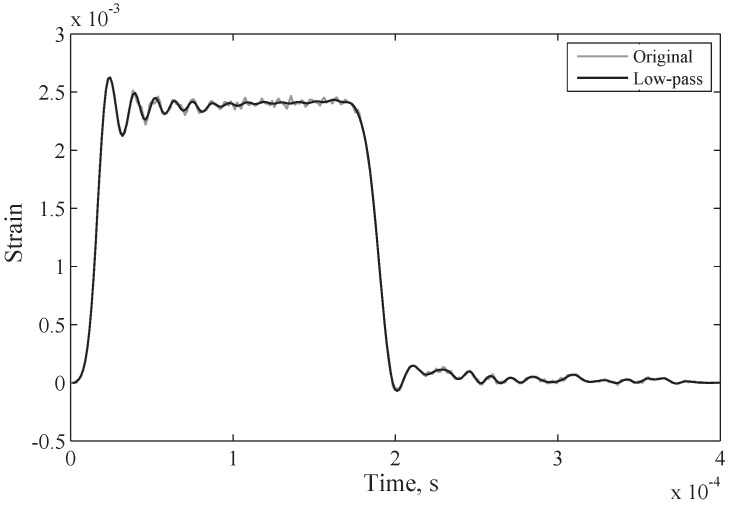
This experimental setup’s incident wave and result of low-pass filtering of frequencies above 94 kHz [7].

In Figure 10, the power at these frequencies is orders of magnitude smaller, and so little information is lost during filtering.

### 4.4. Operation of dispersion.py

When the option for dispersion correction is selected in SHPB_Processing.py, the open-source Python algorithm dispersion.py is called as a subroutine during the processing of the SHPB signals collected from testing.

The function dispersion.py was created to automate the application of phase angle and amplitude correction factors generated by dispersion_factors.py, to SHPB pressure bar signals obtained from the experiments, manipulating the frequency components and correcting the effects of dispersion over a specified propagation length.

The programme dispersion_factors.py is a mandatory subroutine of dispersion.py. After isolating the incident, reflected, and transmitted waves, dispersion.py is used to infer the stress and strain at the bar–specimen interface for each wave using the following command, which includes the input and output variables defined in Table 2. Values for ν may be found from mill specification sheets, and $c_{0}$ from the strain measurements of a low-frequency content wave oscillating in a bar of a known length. Alternatively, these can be calculated using an iterative method, such as that developed in Shin [1].

The following command line is necessary to run this algorithm:

dispersion(x,fs,a,c0,E,z)

This subroutine adapts Tyas and Pope’s [10] dispersion correction approach to ensure that the inferred axial stress and strain data accurately depict the specimen behaviour.

The function’s operation can be summarised as follows:

1. FFT is used to convert the strain signal to a frequency domain signal.
2. The frequency components above the $M_{1}$ correction cut-off are removed using an ideal low-pass filter.
3. Below the Nyquist frequency, for each of the remaining components, the following hold:
  (a) The disperion_factors.py function is used to calculate the required phase shift as well as the factors $M_{1}$ and $M_{2}$. To reduce the computation time, this method employs a pre-calculated, normalised look-up table generated by phase_velocity.py.
  (b) The amplitude correction factor $M_{1}$ and the phase angle correction $\theta_{\omega }^{\prime }$ are used to rebuild a dispersion-corrected strain component using the exponential form of the Fourier series shown in Equation (7) as shown below in Equation (8):
    (8) $$z_{\varepsilon }=M_{1}Ae^{i(\theta -\theta_{\omega }^{\prime })}$$
    where A is the original amplitude of the component, and θ is the original phase angle.
  (c) A dispersion-corrected stress component is similarly reconstructed using factors $M_{1}$ and $M_{2}$, as well as phase angle correction $\theta_{\omega }^{\prime }$ as illustrated in Equation (9) below:
    (9) $$z_{\sigma }=M_{1}M_{2}Ae^{i(\theta -\theta_{\omega }^{\prime })}$$
4. Frequency components above the Nyquist frequency are formed by taking the complex conjugate of these adjusted stress and strain components.
5. The frequency domain stress and strain signals are transformed back to the time domain using inverse FFT ifft() from the numpy library and returned as output variables x_strain and x_stress.

These corrected pressure bar stresses and strains are used in SHPB_Processing.py to infer the behaviour of the SHPB specimen.

### 4.5. Operation of dispersion_factors.py

The Python algorithm, dispersion_factors.py, is a subroutine of the programme dispersion.py. The dispersion factors utilised in this script are calculated using the algorithm phase_velocity.py, with a Poisson’s ratio of 0.29, which is based on the property of the Hopkinson bars used for testing in this case.

Afterwards, dispersion_factors.py loads the four dispersion factor files, m1, m2, norm_freqs and v_ratios, before calculating the amplitude and phase angle corrections required to account for the dispersion at a specific frequency.

The following command line is necessary to run this algorithm, with details of the input and output variables outlined in Table 3:

dispersion_factors(f,a,c0,z)

The corrected angle_mod and m1 and m2 factors are then used in dispersion.py to apply the appropriate signal phase shift to obtain the adjusted strain and stress. It was inspired by a MATLAB script created by Barr [19].

### 4.6. Operation of phase_velocity.py

Phase_velocity.py, is an independent open-source function available on GitHub and ORDA [20]. Its objective is to find the first root of Bancroft’s equation [6] using the bisection method, for a defined Poisson’s ratio, and over a defined range of normalised wavelength (d/L). The result is normalised phase velocity $c_{p}$/$c_{0}$, which corresponds to the first mode of propagation for longitudinal waves in an elastic cylindrical bar.

Normalised wavelengths are also converted to normalised frequency, *f*a/$c_{0}$. Normalised phase velocities are then used to calculate Tyas and Wilson’s [11] factors $M_{1}$ and $M_{1}$, which account for wavelength-dependent radial variations in the strain and Young’s modulus, respectively.

The following command line is necessary to run this algorithm, with details of the input and output variables outlined in Table 4:

phase_velocity(nu,l_ratios)

The factors m1, m2, norm_freqs and v_ratios are then used in dispersion_factors.py and dispersion.py by association to carry out the dispersion correction of the acquired SHPB signals as a functionality of the main processing script SHPB_Processing.py. It was inspired by a MATLAB script created by Barr [21].

## 5. Practical Applications

### 5.1. SHPB Testing

**Figure 11:**
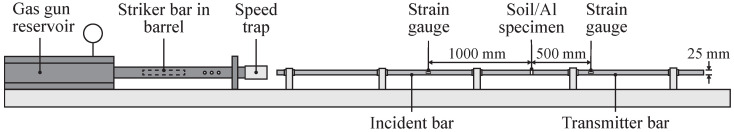
Schematic diagram of the SHPB setup for testing.

A split-Hopkinson pressure bar apparatus consisting of two stainless-steel pressure bars, incident and transmitted bars, was utilised for testing (Figure 11). The gauge locations on the incident and transmitter bars required to process the data were placed at a distance of 1000 mm and 500 mm, respectively, from the sample front and back interfaces. On each bar, a pair of Kyowa KSP-2-120-E4 semiconductor strain gauges was used to record the signals.

### 5.2. Kaolin Clay

A 25 mm kaolin clay sample was tested using a SHPB apparatus configuration detailed in Section 5.1 under unconfined conditions. The sample had an initial length of 5.357 mm, a mass of 4.466 g and a dry mass of 3.167 g. The raw signal data for the incident and transmitter bars were recorded on channels 7 and 8. The incident and transmitter bars were amplified by a factor of 10 and 100, respectively. Dispersion correction was applied. The signals were aligned at the start, and the speed of the striker bar was measured. The data were processed using SHPB_Processing.py with the following command line:

SHPB_Processing(csvfile,[5.357,4.466,3.167],‘None’,[7,8,5],

      [10,100,1],True,‘start’,True)

### 5.3. Sand

A 25 mm medium sand sample was tested using a SHPB apparatus configuration detailed in Section 5.1 under confined conditions. The sample had an initial length of 4.726 mm, a mass of 3.50 g and a dry mass of 3.50 g. The raw signal data for the incident and transmitter bars were recorded on channels 1 and 2. The raw signal for the confining ring was measured on channel 3. The incident and transmitter bars were amplified by a factor of 10. Dispersion correction was applied. The signals were aligned at the start, and the speed of the striker bar fired by the gas gun was measured. The data were processed using SHPB_Processing.py with the following command line:

SHPB_Processing(csvfile,[4.726,3.50,3.50],‘Ring’,[1,2,3],

      [10,10,1],True,‘start’,True)

### 5.4. Aluminium

A 12 mm aluminium sample was tested using the SHPB apparatus configuration detailed in Section 5.1 under unconfined conditions. The sample had an initial length of 5.000 mm, a mass of 1.530 g and a dry mass of 1.530 g. The raw signal data of the incident bars were recorded on channels 1 and 2. The incident and transmitter bars were amplified by a factor of 1. Dispersion correction was applied. The signals were aligned at the start, and the speed of the striker bar was not measured. The data were processed using SHPB_Processing.py with the following command line:

SHPB_Processing(csvfile,[5.000,1.530,1.530],‘None’,[1,2],

      [1,1],True,‘start’,True)

### 5.5. Comparative Analysis of the SHPB Tested Scenarios

With all three SHPB tests processed with the algorithm SHPB_Processing.py, its capabilities are evident since different materials with distinctly different behaviours were run successfully. In each case, the dispersion correction adds 1000 mm of wave propagation to the incident wave, and removes 1000 mm of wave propagation from the reflected wave, taking the frequency dependence of phase velocity into account as described above.

**Figure 12:**
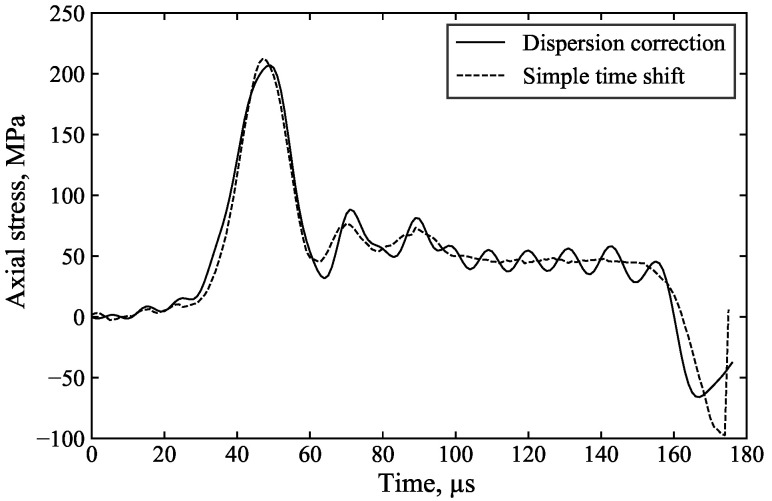
Dispersion vs. time shift analysis of front stress in unconfined SHPB test on kaolin clay.

**Figure 13:**
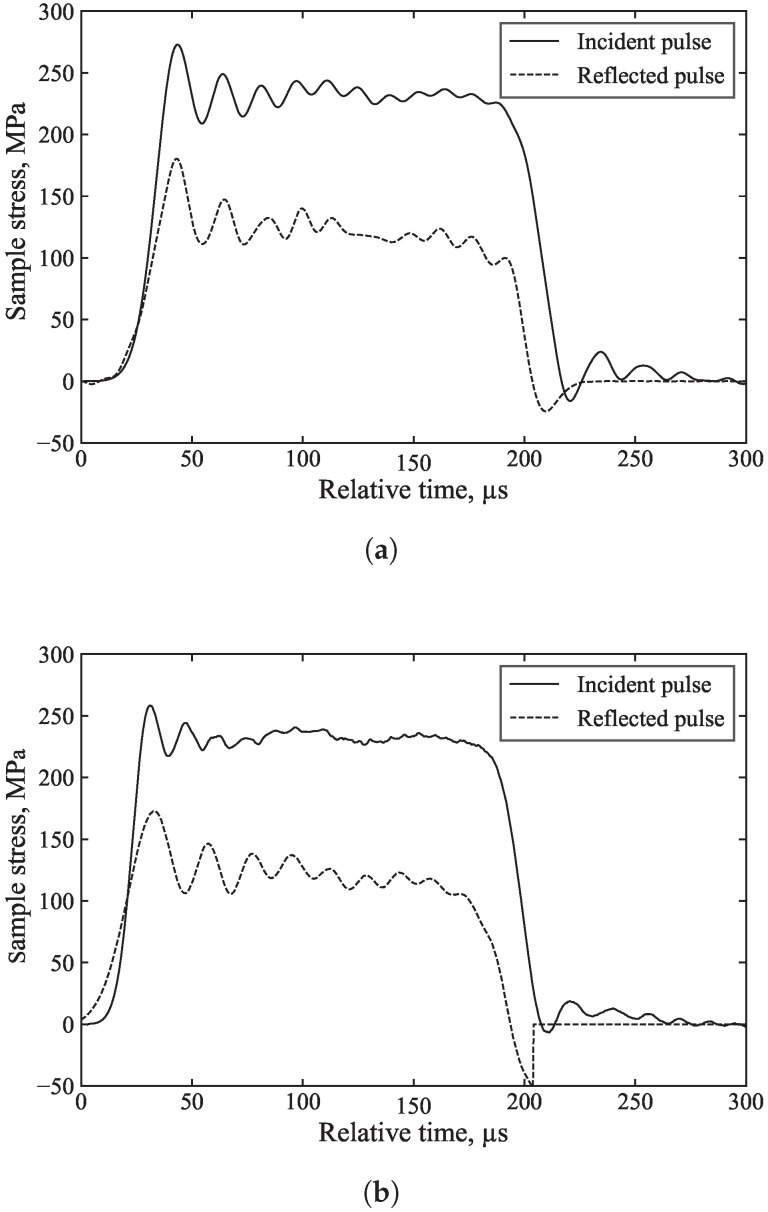

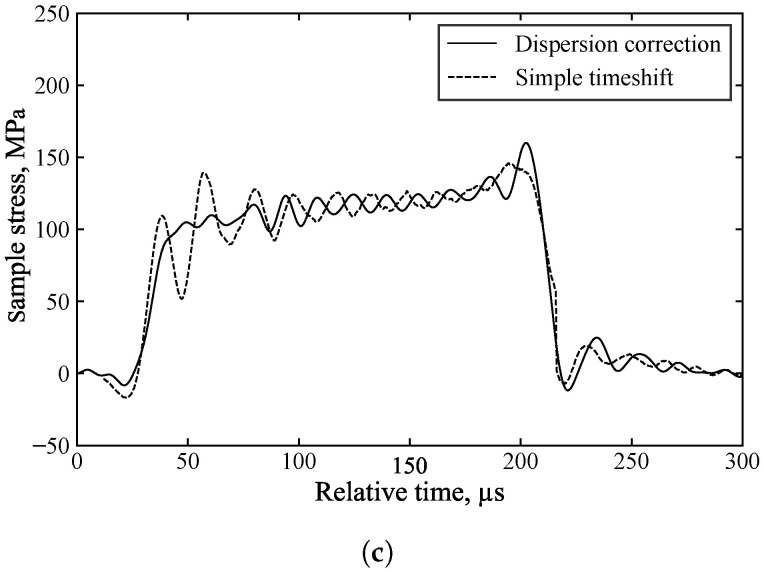
Processed results of the unconfined SHPB test on aluminium with (**a**) dispersion correction of the incident and reflected pulses, (**b**) simple time shifting of the incident and reflected pulses, and (**c**) dispersion correction vs. simple time shifting of the front stress.

**Figure 14:**
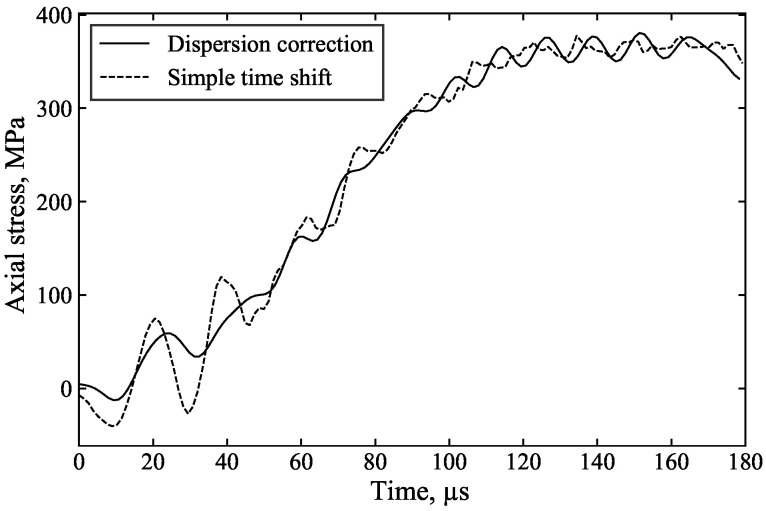
Dispersion vs. time shift analysis of front stress in confined SHPB test on sand.

As the specimen front stress is calculated by the superposition of the incident and reflected waves, the inferred front stress is greatly improved as a result: Figure 12, Figure 13 and Figure 14 highlight the benefits of dispersion correction vs. simple time shifting analysis. 

For example, in Figure 13, the measured incident and reflected waves exhibit significant initial oscillations caused by dispersion associated with the rapid stress rise. Dispersion correction (Figure 13a), applied to account for the 1000 mm of travel from the strain gauge to the specimen, ensures that the changing shape of these oscillations is properly accounted for, resulting in their cancellation in the calculation of the specimen front stress (Figure 13c). In contrast, simple time shifting of the signals (Figure 13b) does not account for these changes in shape and position, leading to spurious oscillations in the inferred specimen front stress. This demonstrates that dispersion correction provides more accurate axial stress data and a clearer understanding of specimen behaviour. The additional processing time required for dispersion correction is minimal, approximately 5 s.

### 5.6. Stress Wave Equilibrium

**Figure 15:**
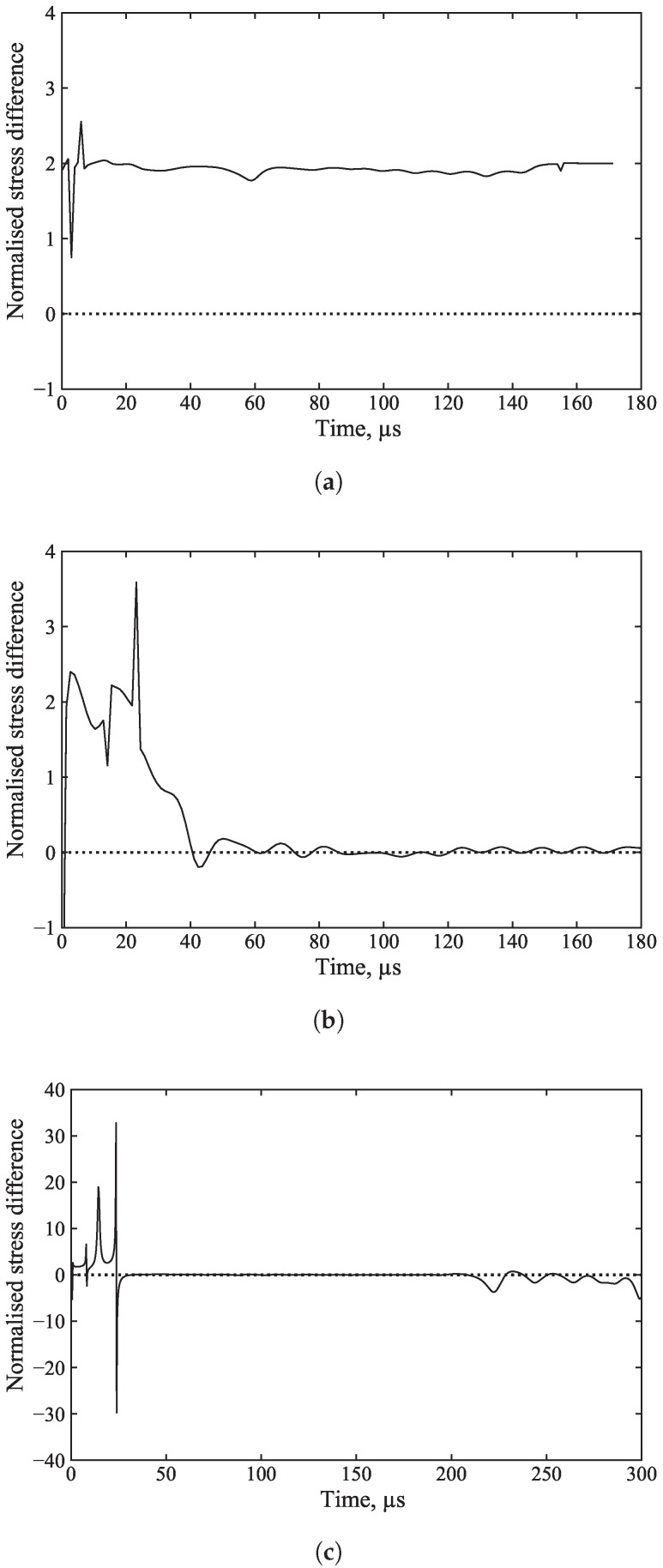
Stress wave difference between front and back stresses, normalised by their mean, for (**a**) an unconfined SHPB test on kaolin clay, (**b**) a confined SHPB test on medium sand, and (**c**) an unconfined SHPB test on aluminium.

The stress difference between the front and back stress normalised by their mean was plotted for all cases, as shown in Figure 15a–c, to demonstrate that even if stress waves do not achieve equilibrium, SHPB_Processing.py still runs successfully and produces accurate results.

In the ideal circumstance, where stress equilibrium is achieved during SHPB testing, the lengths of the pulses detected at the specimen’s front and back bar interfaces (i.e., front and back stresses) will be the same. However, there are instances, more commonly in cases where the stress wave does not fully propagate through the specimen, causing a considerable portion to propagate laterally.

Stress equilibrium during a SHPB test can be represented by Equation (10), provided that the deformation of the specimen is uniform and that the axial propagation of the stress wave has been taken into account:

(10) $$\varepsilon_{i}\left(t\right)=\varepsilon_{r}\left(t\right)+\varepsilon_{t}\left(t\right)$$

The ability in SHPB_Processing.py to set an alignment to manage the front and back stresses means this function is able to account for cases where stress equilibrium may not be fully obtained but an estimation of the axial stress can still be determined, though with the caveat that it should be coupled with further experimental testing or numerical modelling in order to be utilised for material characterisation.

## 6. Discussion

The algorithm SHPB_Processing.py is the main function which performs data processing of the signals obtained from the SHPB tests. It is composed of subroutine dispersion.py, which carries out the dispersion correction of the signals acquired from the experiments. Another function titled dispersion_factors.py is used in this subroutine.

The programme dispersion_factors.py reformats the dispersion correction factors computed by phase_velocity.py. These factors are determined using the Poisson’s ratio of the cylindrical bar used during SHPB experiments. They can easily be obtained for any material. This function and associated subroutines can be used independently.

As seen in the section of this paper devoted to the script, it has a broad range of capabilities, including confinement, signal amplification, dispersion correction or simple time shifting, signal alignment, striker speed measurement, test log monitoring and data saving. Furthermore, because the input and output signals in the Hopkinson pressure bars are mapped independently, the script runs effectively regardless of whether stress wave equilibrium is attained or not. Since the code focuses on SHPB data processing, as the name suggests, it makes the procedure more efficient.

The script’s practical applications were evaluated using SHPB tests with aluminium, kaolin clay and sand samples. An unconfined aluminium sample, an unconfined kaolin clay sample, and a confined medium sand sample were tested with a SHPB apparatus. Most of the script’s functionalities were employed to examine these SHPB experiments, most notably, dispersion.py, which contrasted dispersion corrected and simple time shift results, demonstrating the importance of this script for reliable data analysis.

As demonstrated in the current work, practical applications of SHPB_Processing.py on aluminium, kaolin clay and sand sample data collected from SHPB tests were carried out to illustrate its efficiency, accuracy and broad range of application.

Two of the algorithm’s three confinement possibilities were tested, confined and unconfined SHPB experiments, as seen in the practical application section. A SHPB test using a partial lateral confinement apparatus would be extremely valuable for testing data processing quality. This programme has the advantage of working under various testing conditions, regardless of whether stress wave equilibrium is attained during SHPB testing.

The code was run on a SHPB setup with stainless-steel pressure bars (Poisson’s ratio of 0.29). Yet, SHPB testing with aluminium or polymer bars, which also require dispersion correction, would be extremely valuable to study the script’s performance.

## 7. Conclusions

The essential theory behind dispersion correction and its importance for SHPB experiments were thoroughly investigated. To address this, an invaluable computational tool was created, SHPB_Processing.py, with independent subroutines to complement the script’s already extensive array of functionalities. Practical applications of this function on SHPB tests conducted on aluminium, kaolin clay, and sand samples demonstrate the improved quality of the results, illustrating the immense potential of this open-source algorithm for future applications.

In conclusion, this study underscores the pivotal role of split-Hopkinson pressure bar (SHPB) testing in advancing geotechnical research. By addressing wave dispersion effects and integrating a robust correction algorithm, the proposed approach significantly enhances the accuracy and reliability of stress analysis in soils and other materials. These findings emphasise the value of SHPB testing in understanding dynamic material behaviour, offering crucial insights for geotechnical applications.

## 8. Code Availability

The algorithms developed in this paper are open-source and accessible on GitHub and ORDA at the following links:

- gauge_factor.py: GitHub and ORDA
- phase_velocity.py: GitHub and ORDA
- SHPB_Processing.py: GitHub and ORDA
- dispersion.py&dispersion_factors.py: GitHub and ORDA

## Figures and Tables

**Table 1:** Input and output variables used in SHPB_Processing.py.

**Inputs**	**Description**
csv_path	File directory containing CSV file with raw test data.
sample_data	Array containing the initial length, mass, and dry mass of the sample, i.e., [initial length, mass,
	dry mass].
confinement	Confinement mechanism applied, i.e., ‘None’, ‘Ring’, or ‘Reservoir’.
signal_channels	Oscilloscope channel numbers used to record raw data,
	i.e., [in_bar_gauge_channel, out_bar_gauge_channel, ring_gauge_channel or reservoir_gauge_channel].
signal_amp	Strain gauge amplification applied to strain gauge measurement,
	i.e., [in_bar_gauge_amp, out_bar_gauge_amp, ring_gauge_amp].
disp_correction	Apply dispersion correction or simple time shift processing for signal data,
	i.e., “True” for dispersion correction using dispersion.py.
alignment	Specify alignment mode for aligning stress waves at sample interfaces,
	i.e., ‘start’ aligns the start of incident and transmitted pulse, ‘end’ aligns the end, and ‘mid’ aligns the median time of the pulse. Integer/float values greater than 1 align the peaks of the incident and transmitted pulse to specific times. Float values between 0 and 1 align the incident and transmitted pulse on a specific fraction of the max value.
speedtrap	Specify speed trap data to determine striker bar velocity, i.e ‘True’ for speed trap velocity
	calculation.
**Outputs**	**Description**
Processed data folder	Folder with all the CSV processed data files, and test log for history monitoring.

**Table 2:** Input and output variables used in dispersion.py.

**Inputs**	**Description**
x	Zero-padded strain signal in time domain (1xN numeric).
fs	Sampling frequency, Hz.
a	Bar radius, m.
c0	One-dimensional wave velocity of the bar, m/s.
E	Young’s modulus of the bar, GPa.
z	Distance to apply correction over, positive in direction of
	propagation, m.
**Outputs**	**Description**
x_strain	Dispersion-corrected strain signal.
x_stress	Dispersion-corrected stress signal, MPa

**Table 3:** Input and output variables used in dispersion_factors.py.

**Inputs**	**Description**
f	Frequency, Hz.
a	Bar radius, m.
c0	One-dimensional wave velocity of the bar, m/s.
z	Distance to apply correction over, m.
**Outputs**	**Description**
angle_mod	Phase angle correction, rad.
m1	Correction for variation in response across bar cross section.
m2	Correction for variation in ratio of axial stress and axial strain
	(dynamic Young’s modulus).

**Table 4:** Input and output variables used in phase_velocity.py.

**Inputs**	**Description**
nu	Poisson’s ratio of bar material used for SHPB tests.
l_ratios	Normalised wavelength range to calculate the first root of
	Bancroft’s(1941) equation [6].
**Outputs**	**Description**
dispersion_factors	Folder which includes 4. pickle files containing the dispersion
	factors m1, m2, norm_freqs and v_ratios.

## Data Availability

The data presented in this study are available upon request from the authors.
